# Enhanced-quantum yield sulfur/nitrogen co-doped fluorescent carbon nanodots produced from biomass *Enteromorpha prolifera*: synthesis, posttreatment, applications and mechanism study

**DOI:** 10.1038/s41598-017-04754-x

**Published:** 2017-07-03

**Authors:** Yuanhong Xu, Dan Li, Mengli Liu, Fushuang Niu, Jingquan Liu, Erkang Wang

**Affiliations:** 1 Center for Micro/Nano Luminescent and Electrochemical Materials, College of Materials Science and Engineering; Institute for Graphene Applied Technology Innovation; Laboratory of Fiber Materials and Modern Textiles, the Growing Base for State Key Laboratory; Collaborative Innovation Center for Marine Biomass Fibers Materials and Textiles of Shandong Province, Qingdao University, Qingdao, 266071 China; 2 State Key Laboratory of Electroanalytical Chemistry, Changchun Institute of Applied Chemistry, Chinese Academy of Sciences, Changchun, Jilin, 130022 China

## Abstract

*Enteromorpha prolifera* (*E. prolifera*), one of the main algae genera for green tide, significantly influences both the coastal ecological environment and seawater quality. How to effectively utilize this waste as reproducible raw resource with credible application mechanism are urgent environmental issues to be solved. Herein, *E. prolifera* was converted to attractive fluorescent carbon nanodots (CNDs) by one-pot green hydrothermal process. The purity and quantum yields for the as-prepared CNDs can be enhanced upon the post-treatment of ethanol sedimentation. The CNDs can be well dispersed in aqueous medium with uniform spherical morphology, narrow size distribution and average size of 2.75 ± 0.12 nm. The ease synthesis and relatively high quantum yields of the CNDs make *E. prolifera* inexpensive benefit to the human and nature, such as applications in efficient cell imaging and fiber staining. Furthermore, it was discovered that the fluorescence intensity of the CNDs can be selectively quenched upon Fe^3+^ addition, which can be used for specific sensitive assay and removal of Fe^3+^ in aqueous medium. More importantly, it was reasonably proposed that the quenching was resulted from the synergistic effects of CNDs aggregation and Fe^3+^-CNDs charge-transfer transitions due to the coordination interactions between Fe^3+^ and the oxygenous groups on the CNDs.

## Introduction

*Enteromorpha prolifera (E. prolifera)*, a green macroalgae, has been attracting special and increasing attention recently, especially since 2007 in China’s Yellow Sea. Its large-scale blooming leads to the outbreak of “green tide”, which could flock to the seaway and the shore, then decay to become waste. It significantly influences not only the coastal ecological environment but also the seawater quality, and moreover, leading to great blow to the local tourism^1, 2^. Therefore, how to reasonably and effectively utilize this algal waste is an urgent environmental issue^3^. Meanwhile, *E. prolifera* belongs to one renewable biomass possessing advantages of higher production, faster growth rate and cost-effective availability^4^. It should be a favorable option to offer abundant feedstock for development of energy and material science. Investigations have been conducted to convert it into bio-fuel or oil^1–6^, reducing sugar^7^ for food, animal feed and chemical fertilizer. *E. prolifera* can also be applied to produce high-surface-area activated carbon through a dry-grind strategy^8^. Some *E. prolifera*-like algal substances such as cyanobacteria and green algae were also suggested to be used for assisting the green transformation of Au(III) to gold nanoparticles^9^ or nanorods^10^ due to the reduction effect of the algal substances. However, little information is available regarding the conversion of *E. prolifera* itself into carbon nanomaterials as well as the corresponding applications to date.

Carbon nanomaterials, especially carbon nanodots (CNDs), have attracted increasing attention recently due to their intrinsic biocompatibility, low toxicity, chemical inertness and valuable photoluminescence^11–14^. They have great promise for a variety of practical applications, such as bio-chemical assays, photocatalysis and cell imaging^11, 15, 16^. Particularly, heteroatoms (N or S or B) doped CNDs^17, 18^ were paid considerable attention since they not only maintain most advantages of blank CNDs, but also efficiently improve their properties such as avoiding their self-quenching and improving the PL quantum yield^19^. Despite recent amazing advances in the preparation of CNDs, the use of stringent reaction conditions, toxic or high-cost precursors and tedious post synthetic steps for surface passivation often complicated and hindered their wide applications^15^. Accordingly, it is highly attractive to produce CNDs through green synthetic routes using natural, green carbon precursors such as plant leaves^15, 16^, grass^12^, *Bombyx mori* silk^19^, apple juice^11^ since they are inexpensive, clean, nontoxic and easily accessible.

However, there are still several shortcomings or unresolved problems existing in using the natural green precursors for CNDs preparation. Firstly, the complicated composition (e.g. carbohydrate, fatty acid, minerals) of the biomass, which was seldom discussed till now, would lead to unexpected complex by-products in the CNDs. Secondly, the prepared CNDs usually retained relatively low quantum yields (QYs) (<8%)^11, 12, 15, 20–22^. To enhance the QYs, besides the hydrothermal treatment or pyrolysis of the precursors, additional plasma and microwave treatment are usually needed, which require special instruments, complicated synthetic procedure and increased fabrication cost^16, 23^. CNDs with higher QYs (>10%) can be also been reported by Tyagi and Liang *et al*. using lemon peel^24^ or gelatin^25^ as carbon resources without complex purification. Thus the quality of the CNDs using biomass as precursors is determined by the types of the biomass, synthesis methods and post-treatment techniques. Last but not the least, although the as-prepared CNDs can be widely used in various applications such as specific sensing of Cu^2+^ or Fe^3+^, detailed mechanism and further practical applications are scarcely presented^12, 16^. Therefore, there remains broad research space for this nascent topic to explore more renewable green carbon sources, produce more heteroatoms-doped CNDs with highly improved quality, and broaden the applications of CNDs, especially make contributions for the growingly human concerns nowadays such as environmental protection and disease diagnosis^16^.

Herein, *E. prolifera* was converted to fluorescent carbon nanomaterials, especially sulfur/nitrogen codoped CNDs, by a one-pot green hydrothermal method. Effects of the temperature and reaction time on the yield of CNDs were investigated. Simple post-treatment way of ethanol sedimentation was proposed to decrease the unexpected by-products in the CNDs products. Techniques including UV-vis spectroscopy, fluorescence spectroscopy, X-ray photoelectron spectroscopy (XPS), Fourier transform infrared (FTIR) spectroscopy, transmission electron microscopy (TEM) and atomic force microscopy (AFM) were applied for the characterization of the as-prepared CNDs. A credible mechanism for PL quenching of CNDs by ferric ion Fe^3+^ was proposed based on the characterization results. Based on the fact that the of CNDs of PL can be quenched by Fe^3+^, not only specific and sensitive Fe^3+^ sensing was achieved, the removal of excessive Fe^3+^ in aqueous medium was also proposed. These CNDs have also been successfully applied in efficient cell imaging and as fluorescent inks.

## Results and Discussion

### Conversion of *E. prolifera* into CNDs and corresponding characterizations

**Figure 1:**
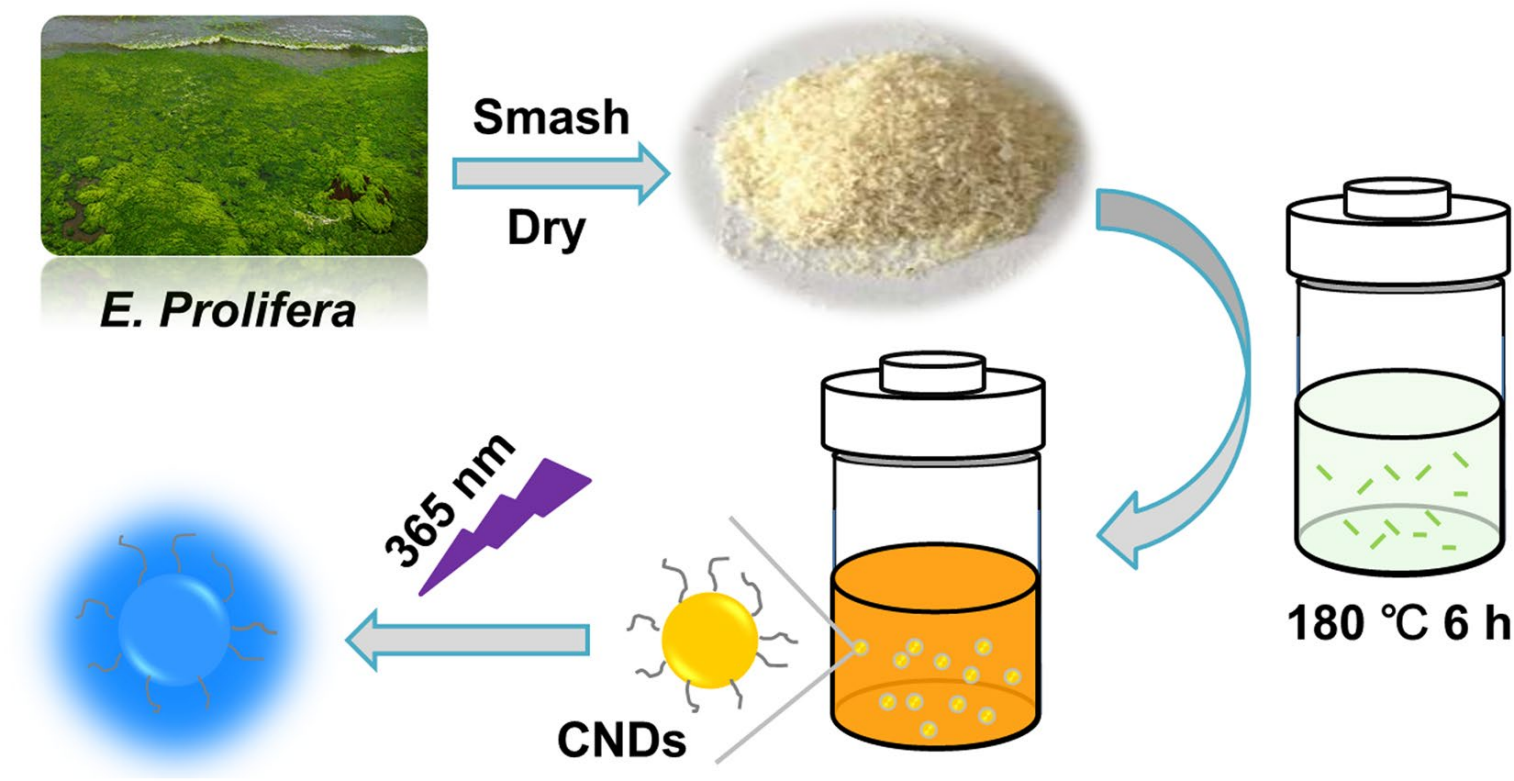
Schematic illustration for CNDs generation via hydrothermal treatment of *E. prolifera* at 180 °C for 6 h and the colour of the produced dispersion under daylight and UV light illumination at 365 nm.

*E. prolifera* belongs to one renewable biomass possessing advantages of higher production, faster growth rate, easily availability and low cost. It is supposed to be an excellent natural and green precursor for synthesizing carbon nanomaterials without the aid of any additional passivating agent. As schemed in Fig. 1, the hydrothermal treatment of dry smashed *E. prolifera* leads to a yellow or light brown dispersion, which emits bright blue light under UV irradiation at 365 nm. The hydrothermal temperature, reaction time and post-purification methods were further optimized for the preparation of carbon nanoparticles, respectively.

Firstly, it was noticed that although the synthesized suspension showed excellent optical properties under UV exposure at 365 nm, TEM images with clear and well dispersed nanodots were hardly observed indicating the impurity surrounding the CNDs. It should be because of the complex components such as crude protein, lipid, and inorganic salts in the biomass *E. prolifera*. This impurity should be one of the reasons for the low QYs (1.2–7.1%) of the CNDs prepared via hydrothermal treatment of varied other biomass such as coriander leaves^15^, grass^12^, apple juice^11^, potato^20^, watermelon peel^21^, oats^23^, etc. (Table 1). Thus, besides the common purification methods such as filtration, dialysis and centrifugation^11, 20^, other post-treatment techniques such as plasma and microwave have to be applied to reach relatively higher QYs^12^.

**Table 1:** Comparison of QYs and applications among varied CNDs from different biomass as carbon sources prepared by hydrothermal treatment at different conditions and post-treatment methods.

Biomass	Reaction conditions	Post-treatment	QYs	Applications	Refs
Coriander leaves	240 °C for 4 h	Filtration	6.48%	Antioxidants, sensors and bioimaging	15
Grass	180 °C for 3 h	Centrifugation	4.2%	Detection of Cu^2+^ in real water samples	12
Apple juice	150 °C for 12 h	Filtration, centrifugation, dichloromethane washing, dialysis	4.27%	Bioimaging	11
*Solanum tuberosum*	170 °C for 12 h	Filtration, centrifugation, dichloromethane washing, dialysis	6.14%	Cell imaging	20
Watermelon peel	220 °C for 2 h	Filtration, centrifugation, dialysis	7.1%	Bioimaging	21
OAts	400 °C for 2 h	Centrifugation, PL enhancement by further microwave treatment	1.2%, 3.0%	Detection of Al^3+^ ions and pH values, cellular imaging	23
coffee grounds	300 °C for 2 h	Filtration, centrifugation	3.8%	Cell imaging and detection of angiotensin I and insulin	22
*E. prolifera*	180 °C for 3 h	Centrifugation, ethanol sedimentation	10.1%, 12.3%	Bioimaging, fiber staining, specific Fe^3+^ detection	This work

Herein, ethanol sedimentation was tested to remove the inorganic salts and possible organic species^16^ from the CNDs suspension. Upon adding ethanol into the as-synthesized CNDs, a large amount of sediments appeared, which was then removed by centrifugation. Several cycles of ethanol addition and centrifugation were repeated until no sediments appeared. The ethanol in the resultant CNDs suspension was evaporated under 30 °C to obtain pure solid-state CNDs. The as-purified CNDs were suspended in water to obtain the suspensions with required concentrations for further characterization and use. The QYs for the CNDs prepared before and after the ethanol sedimentation reached 10.1% and 12.3%, respectively, which were higher than most of those obtained from other biomass as carbon sources for CNDs generation. Table 1 shows the comparison of QYs among different CNDs obtained from varied biomass as carbon sources via hydrothermal treatment at different conditions and post-treatment methods. Based on the comparison, it can be observed that not only the *E. prolifera* is a favorable biomass carbon source for CNDs production, but also the ethanol sedimentation is one efficient way for CNDs purification and QYs enhancement. Furthermore, due to the low boiling point of ethanol, solvent in the CNDs suspension obtained after ethanol sedimentation can be removed upon volatilization, which is favorable for the preparation of the solid-state CNDs, thus facilitated the following accurate weight quantification for the further applications.

**Figure 2:**
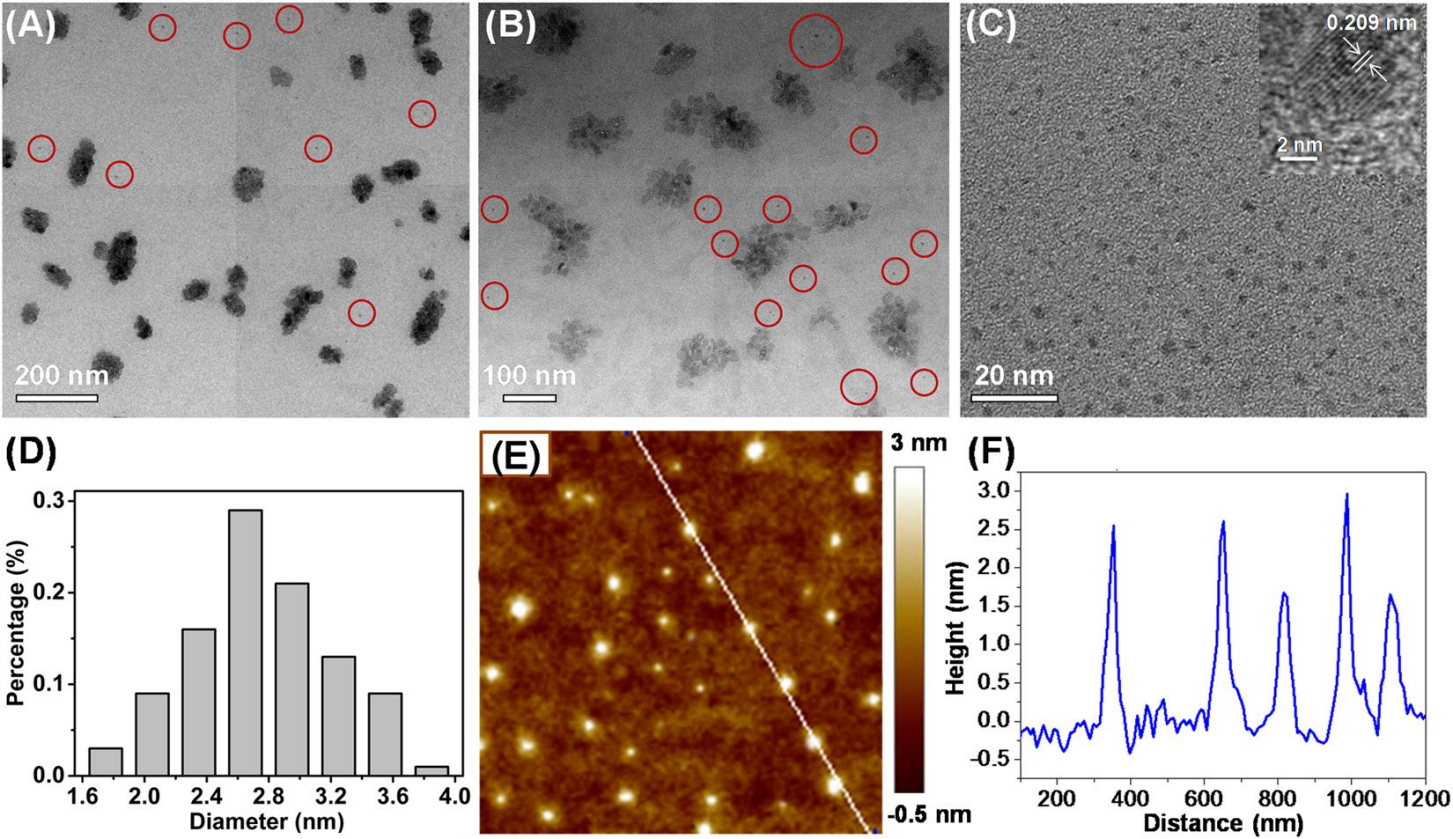
Representative TEM images of the CNDs dispersion, which were generated via hydrothermal treatment of *E. prolifera* at 150 °C for 3 h (**A**), 180 °C for 3 h (**B**), 180 °C for 6 h (**C**), respectively. HRTEM image (inset of **C**), the particle size distribution calculated based on TEM image (**E**), AFM topography image on silicon substrate, (**E**) and corresponding height profile, (**F**) of the CNDs prepared via hydrothermal treatment of *E. prolifera* at 180 °C for 6 h.

Figure 2A–C shows the transmission electron microscope (TEM) images of the products prepared via hydrothermal treatment of *E. prolifera* at different reaction temperature and time. As shown in Fig. 2A, relatively lower temperature (150 °C) and shorter reaction time (3 h) mainly resulted in aggregated carbon nanoparticles ranging from 30 to 140 nm, accompanied by relatively fewer scattered CNDs as red-circled. Increasing temperature to 180 °C for the same reaction time (3 h) can reduce the aggregation degree of the carbon nanoparticles and increase the formation of CNDs (red-circled) as indicated in Fig. 2B. Furthermore, products prepared at 180 °C for 6 h were all composed of CNDs well-separated each other. Well-resolved lattice fringes with an interplanar spacing of 0.209 nm were observed in the crystalline structures of the CNDs as evidenced by high-resolution TEM (HRTEM) measurement (inset of Fig. 2C), which agrees well with the (102) diffraction planes of sp^2^ graphitic carbon and is close to the (103) diffraction plane of diamond-like (sp^3^) carbon^26, 27^. The results suggest that the proposed CNDs are mainly composed of sp^2^ graphitic carbons with sp^3^ carbon defects^16^. The corresponding particle size distribution histograms (Fig. 2D) indicate that these nanodots have average diameter of 2.75 ± 0.12 nm as calculated from counting over 100 particles. The CNDs were also characterized using AFM measurements. The AFM images (Fig. 2E) with associated height profiles (Fig. 2F) revealed that the as-prepared CNDs ranged between 1.5 and 3.0 nm in diameter. The combination of TEM and AFM results show that the CNDs appear as spherical particles and have uniform dispersion without apparent aggregation. In addition, we noted that further increasing the hydrothermal temperature to 200 °C or the reaction time to 10 h did not produce greater amounts of CNDs, which was supported by the slight fluorescence decrease for the CNDs solutions prepared with the same amount of starting materials. As a result, 180 °C and 6 h were chosen as the optimized hydrothermal reaction conditions for the preparation of CNDs.

**Figure 3:**
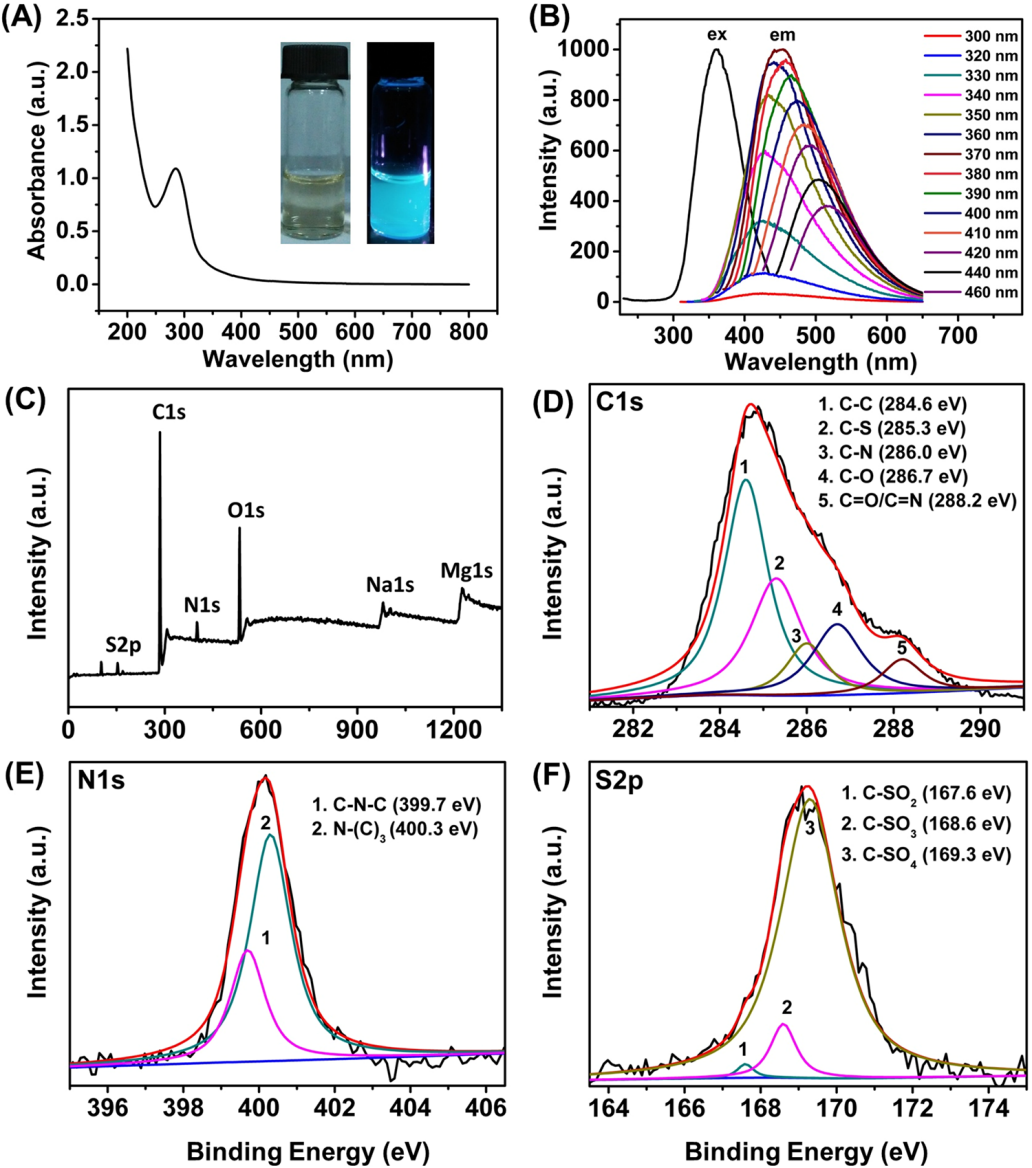
UV-vis absorption spectrum of CNDs. Photographs of as-prepared CNDs suspension under room light (inset, left) and upon irradiation with 365 nm UV light (inset, right). (**B**) Emission (em) spectra at progressively increasing excitation wavelengths from 300 nm to 460 nm and excitation (ex) spectrum of the CNDs suspensions. XPS spectra of the as-prepared CNDs: (**C**) Survey XPS spectrum; Narrow scan spectra of (**D**) C1s; (**E**) N1s; (**F**) S2p, respectively.

The optical properties of the CNDs were investigated as indicated by the typical UV-Vis spectrum (Fig. 3A) and fluorescence spectra (Fig. 3B), respectively. The characteristic absorption peak at 286 nm in the UV-Vis absorption should be ascribed to π–π* transition of C = C bonds within the CNDs, which is consistent with some other previous reports^19, 23^. As shown in the inset of Fig. 3A, the diluted suspension of CNDs exhibited light-yellow colour under daylight with excellent aqueous dispersibility while emitted distinct blue luminescence under exposure to 365 nm UV irradiation. This phenomenon confirmed that the CNDs possessed strong PL properties. In addition, the fluorescence spectra depicted an excitation dependent behaviour of the CNDs (Fig. 3B). With the increasing excitation wavelength from 300 nm to 460 nm, the maximum emission shifted from 417 nm to 517 nm along with a concurrent first increase and then decrease in emission intensity (Fig. 3B). Maximum emission was observed at 450 nm when excited at 370 nm. The suspension of the CNDs showed good stability as confirmed by the almost unvaried fluorescence intensity as the initial one even after two-month storage in air at room temperature.

The surface composition and element analysis of as-prepared CNDs were characterized by X-ray photoelectron spectroscopy (XPS). The XPS spectrum (Fig. 3C) shows three peaks at 169.2, 284.8, 399.9, and 532.4 eV which are corresponding to S2p C1s, N1s, and O1s, respectively. Other two peaks at 980.0 and 1227.1 eV associated with Na1s and Mg1s are also observed. XPS results indicate that the CNDs are mainly composed of C, N, O as well as limited amount of S, Na and Mg elements, which may come from the inorganic salt in the raw materials of *E. prolifera*. The C1s spectrum indicated five types of carbon bonds: C-C (284.6 eV), C-S (285.3), C-N (286.0 eV), C-O (286.7 eV) and C = O/C = N (288.2 eV), respectively (Fig. 3D). Two peaks at 399.7 and 400.3 eV in the N1s spectrum (Fig. 3E) should be ascribed to the C–N–C, N–(C)_3_ bands, respectively^16^. The S2p spectrum (Fig. 3F) exhibits three peaks at 167.6, 168.6 and 169.3 eV, which indicated the existence of C-SO_x_ (x = 1, 2, 3) groups on the CNDs. According to the results obtained via the dynamic light scattering (DLS) measurement, the average zeta potential of the as-prepared CNDs suspension was −2.57 mV, indicating the negative surface charge of the CNDs^28^. All these results confirmed the presence of oxygenous groups such as hydroxyl, carbonyl and carboxylic acid groups and the successful doping of the N, S elements in the as-prepared CNDs.

**Figure 4:**
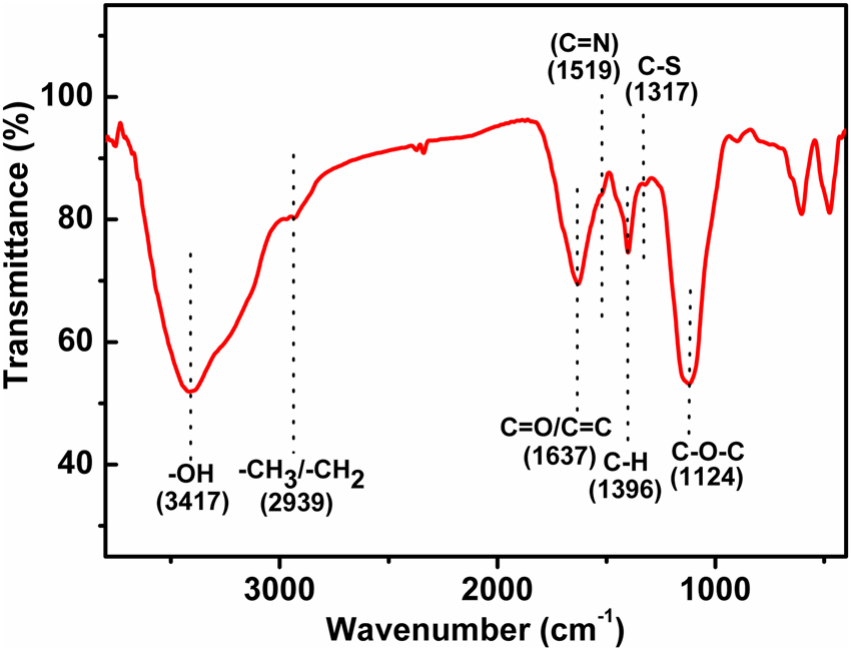
FTIR Spectra of the as-prepared CNDs.

FTIR spectrum was further used to investigate the surface functional groups presenting on the CNDs (Fig. 4). The peak at 3417 cm^−1^ should be ascribed to the O-H stretching vibration. The peak at 1637 cm^−1^ was attributed to the C = O and C = C stretching in the conjugated structure. The peaks at 2939 and 1396 cm^−1^ were related to the C-H bond stretching vibrations. The characteristic stretching band of the C-O-C bond was observed at 1398 cm^−1^. In addition, the peak at 1317 cm^−1^ was due to the stretching vibration of C-S. The FTIR results again verified the abundance of oxygen-containing groups on the surface of the as-prepared CNDs, which made the CNDs well dispersed in aqueous medium. Furthermore, the successful doping of N and S in the CNDs was also confirmed, which should result in the relatively higher QY and the high stability of the CNDs. Both the XPS and FTIR results also confirmed *E. prolifera* was a widespread, easily available, low-cost, and green natural carbon sources for large-scale generation of CNDs since it is abundant with carbon, nitrogen, oxygen and some sulfur elements owing to the existence of crude protein, lipid, amino acids, linolenic acid, etc. in *E. prolifera* ^1, 3^.

### Applications of the as-synthesized CNDs and the proposed mechanism

Based on the attractive features including the nano-size, and superior optical performance, the as-prepared CNDs were supposed to be a valuable fluorescent probes in many applications such as staining, bioimaging and bio-/chem- sensing. Since various pH environments are often encountered in practical applications, the influence of different pH on the fluorescence intensity of the CNDs was investigated. It was observed that the intensity of the CNDs was lightly affected by varied pH values ranging from 2 to 11 (Supplementary Fig. S1). This result showed that the as-prepared CNDs possessed high stability in various pH environments, which is favorable for further applications.

**Figure 5:**
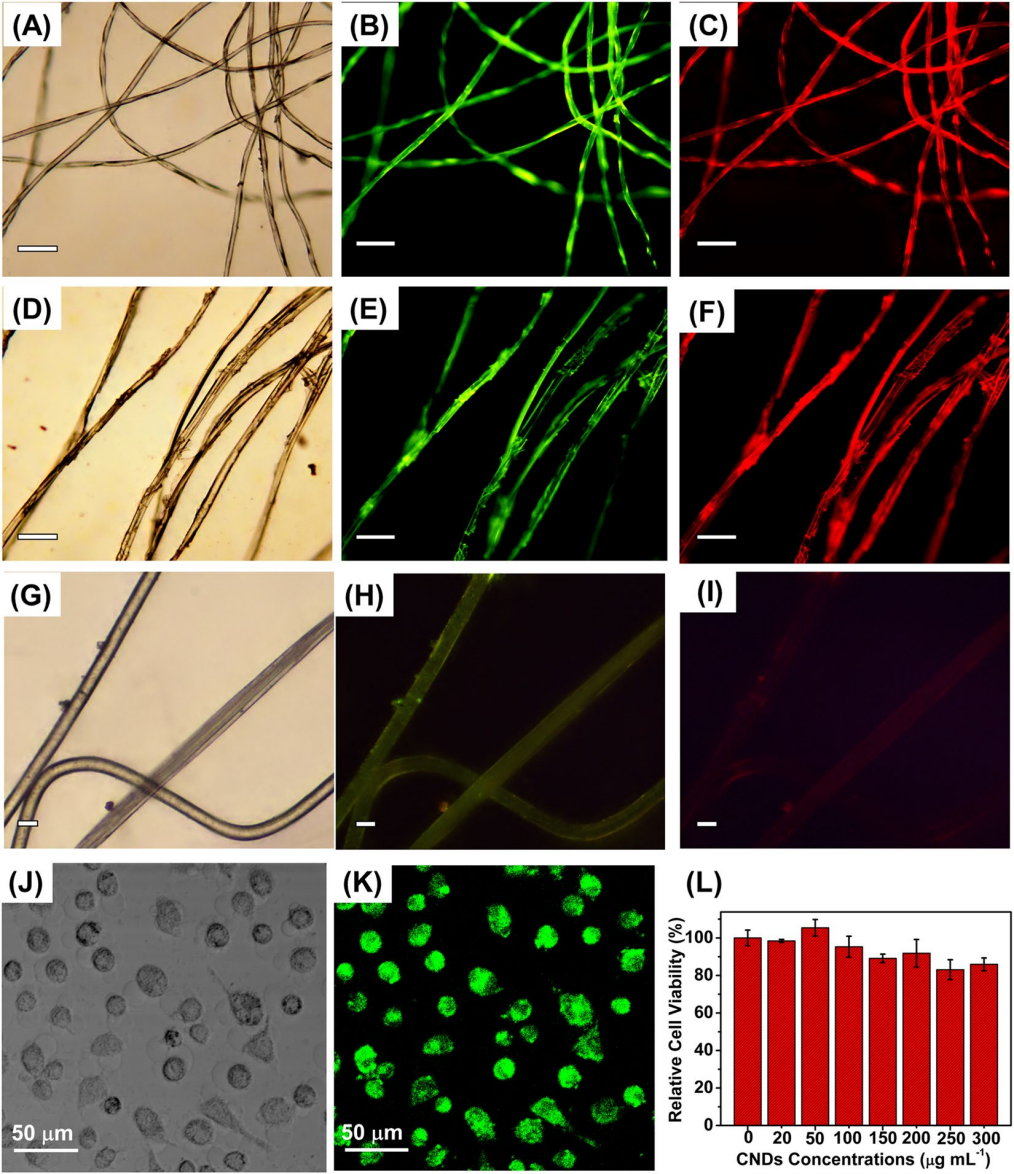
(**A**) Bright field and (**B**,**C**) fluorescence images of cotton fiber stained with the prepared CNDs. (**D**) Bright-field and (**E,F**) fluorescence images of silk fiber stained with the prepared CNDs. (**G**) Bright field and (**H,I**) fluorescence images of cotton fiber without being stained with the prepared CNDs. The fluorescent images were obtained at the excitation wavelengths of (**B,E**) 450–480 nm, and (**C,F**) 510–550 nm. Scale bars of **A–I**: 100 μm. LSCM images of HeLa cells incubated with 100 μg mL^−1^ CNDs for 24 h at 37 ◦C: (**J**) bright field images, (**K**) fluorescence images at excitation wavelength of 488 nm. (**L**) Cell viability of HeLa cells (n = 3, mean ± S.D.) after being incubated with CNDs at various concentrations (0, 20, 50, 100, 150, 200, 250, 300 μg mL^−1^, respectively).

For staining test, the cotton thread and silk fiber were soaked with the aqueous suspension of CNDs, rinsed by water and then dried under air at 50 °C. Accordingly, the cotton thread and silk fiber were coated with CNDs uniformly. As can be seen in Figs 4F–5A, excitation wavelength-dependent fluorescence was observed for the CNDs-stained materials by inverted fluorescence microscope. Both the cotton thread (Fig. 5A–C) and silk fiber (Fig. 5D–F) exhibited bright green and red fluorescence when exposed in blue and green light excitation, respectively. As a control, cotton thread and silk fiber without being stained with the CNDs were also prepared for fluorescence image collection. Taking blank cotton thread as example (Fig. 5G–I), the fluorescence was both very weak upon the exposure to blue (Fig. 5H) and green (Fig. 5I) light excitation under the same fluorimetric parameters. This control experiment excluded the possible influence of the scattering effect on the fluorescence of the stained cotton thread or silk fiber^29^. All these results confirmed the great potential of CNDs for being applied as efficient fluorescent staining reagent.

DLS study revealed the hydrodynamic sizes of the as-prepared CNDs were mainly ranging from 4~8 nm (Fig. S3), which was consistent with the TEM and AFM characterizations. Their small sizes (less than 10 nm) may facilitate the diffusion in cells and the easy cellular uptake of the fluorescent CQDs for efficient bio-imaging^30^. For bioimaging applications, HeLa cells were incubated with the 100 μg mL^−1^ CNDs for 24 h at 37 ^◦^C, followed by image acquisition via LSCM under 488 nm light excitation. As showed in Fig. 5G,H, the HeLa cells incubated with CNDs presented excellent and reproducible morphology with bright green fluorescence. These results confirmed the excellent biocompatibility of the as-fabricated CNDs, which are favorable for being untaken by the cells for efficient bioimaging. Low cytotoxicity is one of the prerequisites for a qualified biomaterial to be employed for cell imaging. The cytotoxicity of the CNDs was then studied with a standard 3-[4,5-dimethylthiazol-2-yl]−2,5-diphenyltetrazolium bromide (MTT) cell proliferation assay. Figure 5I shows the survival rates of the HeLa cells after being incubated with CNDs for 24 h at concentrations of 0, 20, 50, 100, 150, 200, 250, 300 μg mL^−1^, respectively. Through 24-hour incubation, the CNDs at concentration less than 100 μg mL^−1^ exhibited nearly no cytotoxicity to the HeLa cells. The cell viability tended to slightly decrease in a concentration-dependent behavior with the increasing concentrations. Over 85% cell survival rate can be maintained in the culture medium with CNDs even at the maximum tested concentrations of 300 μg mL^−1^. These results confirmed the excellent biocompatibility of the as-prepared CNDs towards the cells. Moreover, as can be seen in Fig. S2, with 2-hour successive irradiation, only slight fading was observed for the green fluorescence brightness. These results indicated that the as-prepared CNDs can be utilized as an excellent fluorescent bioimaging probes owning to their low toxicity, stability, and resistance to photobleaching.

**Figure 6:**
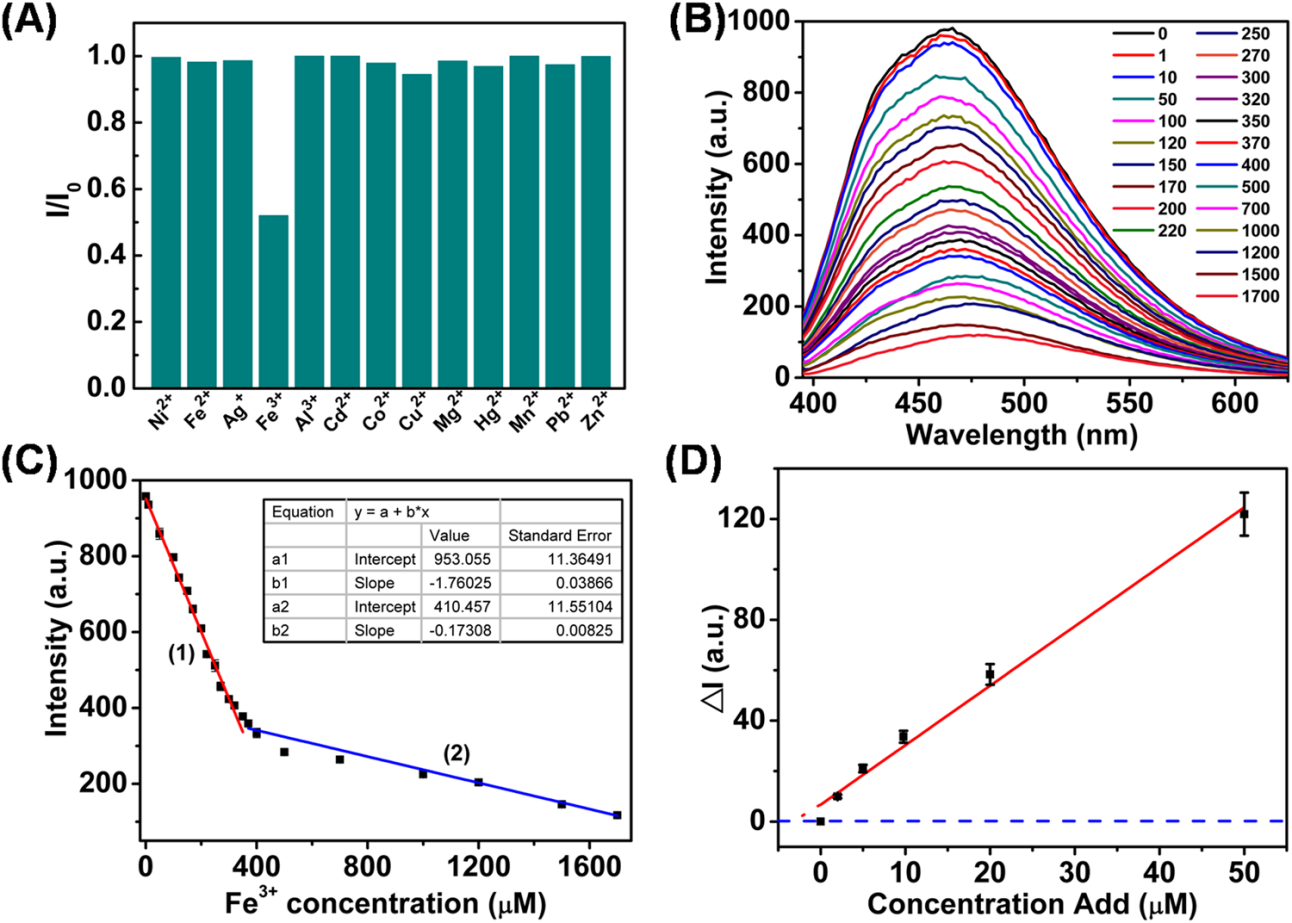
(**A**) Remained percentage of the CNDs PL intensity upon addition of different metal ions all at 250 µM in acidic conditions (pH = 6.0). (**B**) PL intensity of the CNDs in the presence of Fe^3+^ ions at different concentrations from 0 to 1700 µM. (**C**) Plot of PL intensity of CNDs against the Fe^3+^ concentrations. (**D**) Detection of Fe^3+^ ion in tap water using a standard addition method. Excitation at 370 nm was applied. Error bars indicate standard deviations of the means (n = 3).

The effects of different metal ions (all at 250 μM) including Ni^2+^, Fe^2+^, Ag^+^, Fe^3+^, Al^3+^, Cd^2+^, Co^2+^, Cu^2+^, Mg^2+^, Hg^2+^, Mn^2+^, Pb^2+^, Zn^2+^ on the PL intensity of the CNDs at the same concentration were studied. Before the test, the CNDs suspensions were adjusted to acidic conditions (pH = 6.0), in which the metal ions can present in their free forms. The fluorescence intensities of CNDs were significantly quenched in the presence of Fe^3+^, while the other ions showed weak or even negligible quenching effects on the fluorescence intensity (Fig. 6A). Since there are often several metal ions co-existing in practical applications, the fluorescent selectivity of the as-prepared CNDs for metal ions was further evaluated when a mixed solution of metal ions was used. As can be seen in Fig. S4, the mixture of detected ions such as Ni^2+^, Cu^2+^, Co^2+^, Al^3+^, etc. each at 250 µM will not influence the fluorescent sensitivity of the CNDs obviously, while addition of pure Fe^3+^ at the same concentration will bring the intensity of CNDs down significantly. Further addition of the mixed metal ions will not lead to obvious change of the Fe^3+^ containing CNDs, either. These observations indicated that the as-prepared CNDs have highly specific interaction with Fe^3+^, based on which specific assay of Fe^3+^ can be established.

The fluorescence intensities of CNDs upon addition of different concentrations of Fe^3+^ ranging from 0 to 1.7 mM are shown in Fig. 6B. The PL intensity (I) of CNDs against the added Fe^3+^ concentrations (C) was plotted in Fig. 6C. Linear dependences of intensity on the concentration of Fe^3+^ from 1.0 to 370 μM and 400–1700 μM were obtained, with linear regression equations of I = −1.76 C + 953.05 (R = 0.9971) and I = −0.173 C + 410.46 (R = 0.9933), respectively. The limit of detection (LOD) for Fe^3+^ was found to be 0.5 μM (S/N = 3), which is much superior or comparable to those reported in some previous reports^15, 31^. Moreover, the proposed method has the advantages of a wide linear range and a simple preparation procedure^15^.

The applicability of the proposed methods in real sample analysis was evaluated using a standard addition method for detection of Fe^3+^ in tap water (Fig. 6D). The RSD was 0.29% for 3 replicate measurements of the PL intensity of CNDs in the tap water sample spiked with 10 μM Fe^3+^, confirming the good reproducibility and feasibility of the suggested method. Linear relationship (I = 2.36 C + 2.61, R = 0.9952) was achieved and the Fe^3+^ concentration in tap water was measured to be 3.2 ± 0.1 μM (n = 3), a value which is very close to that obtained by atomic absorption spectrophotometery (AAS) analysis (3.0 ± 0.2 μM (n = 3)). The AAS procedure is described in the Supporting Information. The assay shows excellent selectivity, accuracy and reproducibility for Fe^3+^ detection in real samples.

**Figure 7:**
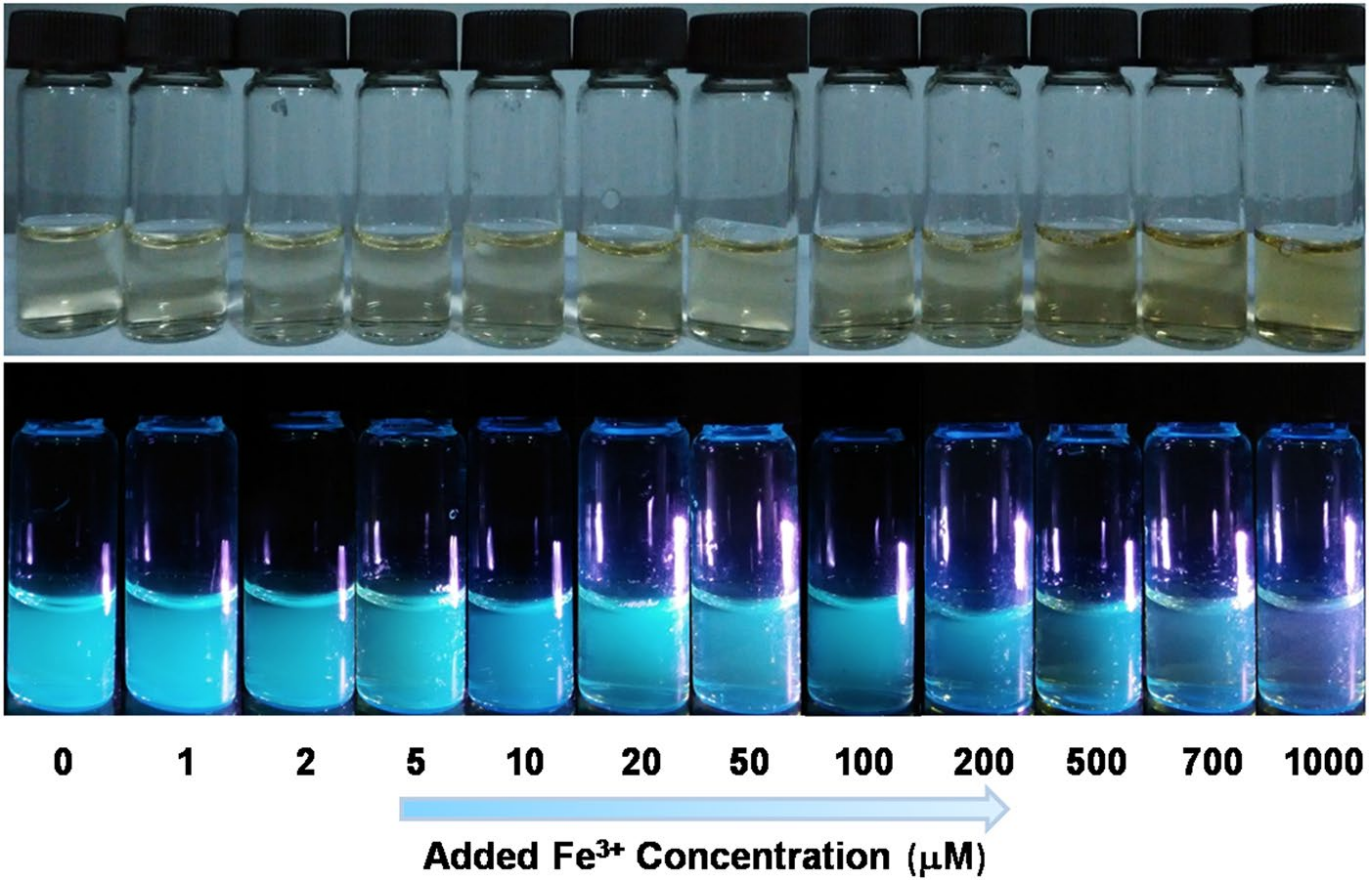
Photographs of as-prepared CNDs suspension upon addition of Fe^3+^ with different concentrations ranging from 0–1000 μM under under daylight (above) and upon irradiation with 365 nm UV light (below).

In addition, photographs of as-prepared CNDs suspension at the same concentration upon addition of different concentrations of Fe^3+^ were collected both under daylight (above) or upon irradiation with 365 nm UV light (below). As shown in Fig. 7, with the increasing concentrations of the added Fe^3+^, the colour of the CNDs suspension under daylight changed from nearly colorless to yellow, while the corresponding blue light under UV irradiation changed from bright to pale finally. These were consistent with the decreasing fluorescence intensities of the CNDs suspensions with the increasing Fe^3+^ concentrations (Fig. 6B and Fig. S5). That is to say, the increasing quenching effect of the PL intensity with increasing Fe^3+^ concentration can be differentiated by naked eyes, which provides possibility for CNDs based-colorimetric detection in the future applications.

**Figure 8:**
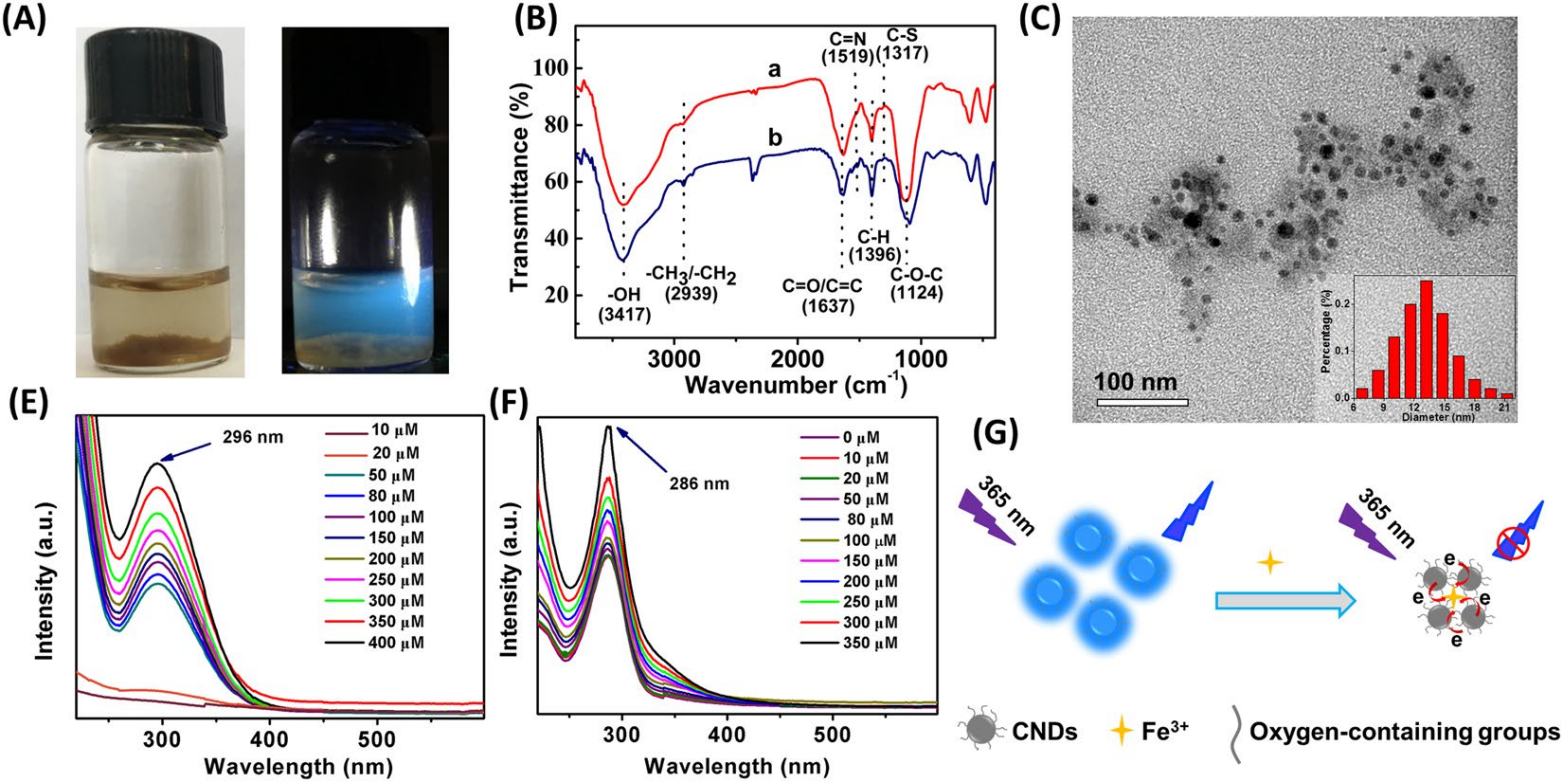
(**A**) The corresponding photographs of the 700 µM Fe^3+^-added CNDs suspension stored for 24 h under daylight (right) and under irradiation with 365 nm UV light (left); (**B**) FTIR spectra of (a) original CNDs suspension; (b) precipitate formed in CNDs suspension upon the addition of 700 µM Fe^3+^ and storage for 24 h. (**C**) TEM image of the supernatant obtained from the CNDs suspension upon addition with 700 µM Fe^3+^ and storage for 24 h. (**E**) UV absorption spectrum of pure Fe^3+^ solution at different concentrations. (**F**) UV absorption spectrum of CNDs in the presence of different concentrations of Fe^3+^. (**G**) Schematic representation of proposed PL quenching mechanism of CNDs by Fe^3+^.

Moreover, when the CNDs suspension was added with 700 µM Fe^3+^ and further stored for 24 h, yellow precipitate was observed (left of Fig. 8A). Under irradiation with 365 nm UV light, the blue light of the supernatant (right of Fig. 8A) was much weaker than that of original CNDs suspension. The yellow precipitate was supposed to be formed due to the coordination interactions between Fe^3+^ and the CNDs, it is supposed that the CNDs can be used as efficient reagent for Fe^3+^ removal in the aqueous medium, which should have great potential in further treatment of waste water containing excessive amounts of Fe^3+^. Meanwhile, no precipitate was formed when the CNDs suspension was added with other ions such as Cu^2+^, Cd^2+^ or Ni^2+^ all at 500 µM for the same incubation time of 24 h. This again confirmed the selective affinity interaction between Fe^3+^ and the oxygen-containing groups on the CNDs as well as the good selectivity for further Fe^3+^ removal applications.

It is supposed that the quenching of CNDs PL intensity by Fe^3+^ should be attributed to the specific affinity interaction between Fe^3+^ and the oxygen-containing functional groups such as hydroxyl or carboxylic groups on the surface of the CNDs. FTIR spectroscopy was carried out in order to identify any changes about the functional groups on the CNDs before and after being treated with Fe^3+^ (Fig. 8B). Compared with the original CNDs suspension (curve a in Fig. 8B), the ratio of the peak at 1637 cm^−1^ being ascribed to the C = O stretching to that at 1396 cm^−1^ resulting from C-H vibration became smaller for the as-formed precipitate upon the addition of Fe^3+^ (curve b in Fig. 8B). The results indicated that the interaction between Fe^3+^ and the oxygen-containing groups on the surface of the CNDs led to relatively decreased free C = O stretching.

Firstly, the interaction between the CNDs and Fe^3+^ could lead to aggregation of the CNDs, which could not only be verified by the visual inspection of the formed precipitate resulting from the addition of Fe^3+^ (Fig. 8A), but also be confirmed by the TEM characterization of the supernatant obtained from the CNDs suspension upon addition with Fe^3+^. As can be seen in Fig. 8C, when Fe^3+^ was added to the CNDs suspension, the average diameter of the resulted CNDs was 13.2 ± 0.3 nm, which was about five times larger than that of the original CNDs before Fe^3+^ addition.

Secondly, since Fe^3+^ has its own absorption peak in the UV-vis range (Fig. 8E), the UV-vis absorbance was also collected for CNDs being added with different concentrations of Fe^3+^ (Fig. 8F). As shown, the UV absorbance peak of pure Fe^3+^ appeared at 296 nm, whose intensity increased with the increasing Fe^3+^ concentrations (Fig. 8E). The UV absorbance peak of pure as-prepared CNDs was found to be 286 nm, whose peak position was unchanged upon further Fe^3+^ addition at various concentrations, but the UV intensity of the CNDs increased with the growing Fe^3+^ concentrations, especially at above 50 µM (Fig. 8F). This phenomenon indicated the energy transfer between the Fe^3+^ and the CNDs, which should be due to the occurrence of charge-transfer transitions between the oxygen-containing functional groups as ligand (electron donor) to Fe^3+^ as coordination center (electron acceptor) under the radiative excitation^32^. Therefore, there is a possibility that part of the excitation energy is itself reduced due to absorbance of Fe^3+^, subsequently resulting in quenching of the CNDs PL intensity.

From the above characterizations, it was supposed that the coordination interactions between Fe^3+^ and the oxygenous groups on the CNDs could result in the aggregation of the CNDs and charge-transfer transitions between Fe^3+^ and the CNDs, the synergistic effects of which led to the specific quenching of CNDs PL intensity by Fe^3+^ (Fig. 8G). When the added Fe^3+^ concentration was lower (e.g. 50 µM), the UV absorbance was much weaker leading to negligible LMCT transitions. As a result, the aggregation effect dominated the quenching of the CNDs PL intensity. While at higher Fe^3+^ concentrations (e.g. above 50 µM), the two effects exerted their influences simultaneously. Due to the higher production, faster growth rate and cost-effective availability of *E. prolifera*, it can be used to synthesize CNDs in mass production or in certain amount according to practical requirement. Corresponding application examples of the as-prepared CNDs in the present work was carried out based on the excellent optical properties of the CNDs and used to confirm the applicability of the CNDs. The huge amount of production of the CNDs from *E. prolifera* provides the possibility to carry out large batches of analytical assays or prepare assay kits in large quantities. In addition, the as-prepared CNDs have great application potentials in other fields such as anti-counterfeiting, energy storage, photoelectric devices.

## Experimental

### Materials

HeLa cells were obtained from the American Type Culture Collection (Manassas, VA) and maintained in DMEM supplemented with 10% standard fetal bovine serum (HyClone Laboratories, UT) at 37 °C and in 5% CO_2_. The 35 mm glass chamber slides were purchased from Hangzhou Sanyou Biotechnology Co. Ltd. (Hangzhou, China). Silver nitrate, mercuric nitrate, MTT, dimethyl sulfoxide (DMSO), and quinine sulfate were purchased from Aladdin Reagent Co., Ltd (Shanghai, China). Aluminum chloride, sodium sulfate, chromium acetate, ferric chloride, copper chloride, cobalt chloride, ferrous chloride, magnesium chloride, lead acetate, manganese chloride, zinc chloride and nickel nitrate were obtained from Tianjin Reagent Co., Ltd (Tianjin, China). Sodium chloride (NaCl), potassium chloride (KCl), disodium hydrogen phosphate (Na_2_HPO_4_), mono potassium phosphate (KH_2_PO_4_) were purchased from Tianjin Guangfu Technology Development Co. Ltd. Ethanol (AR), were purchased from Fuyu Co. Ltd. (Tianjin, China). All chemicals were of analytical grade and were used without further purification. Phosphate buffered saline (PBS, pH 7.4) was prepared containing NaCl (8 g), KCl (0.2 g), Na_2_HPO_4_ (1.44 g), and KH_2_PO_4_ (0.24 g) in 1.0 L solution. *E. prolifera* and seawater were both obtained from the coast of the China’s Yellow Sea in Qingdao. Ultrapure water (18.2 MΩ cm^−1^) from a Milli-Q ultrapure system (Qingdao, China) was used in this study.

### Apparatus

The PL and UV absorption spectra were measured on a Hitachi F-7000 fluorescence spectrophotometer (Tokyo, Japan) and a Mapada UV-1800PC spectrophotometer (Shanghai, China), respectively. The morphology of CNDs was observed on a JEOL Ltd JEM-2010 transmission electron microscope (JEOL Ltd., Japan). AFM images were obtained by using a SPI3800N microscope (Seiko Instruments Inc.) operating in the tapping mode. AFM samples were prepared by depositing the CNDs dispersions on freshly cleaved mica surfaces through a drop-casting method, respectively, and then were left to air-dry. Fourier transform infrared spectra (FTIR) were recorded on a Nicolet 5700 FTIR spectrometer (Thermo Electron Scientific Instruments Corp., USA). X-ray photoelectron spectroscopy (XPS) data were obtained on an ESCALab220i-XL electron spectrometer (VG Scientific, West Sussex, U. K.) using 300 W Al Kα radiation. The fluorescence images of the cells were taken using the LEICA TCS SP2 laser scanning confocal microscope (Leica Microsystems Heidelberg GmbH, Germany) with a 100 × oil immersion objective. The CNDs cultured cells were excited using 488 nm Ar laser. Dynamic light scattering (DLS) data were obtained on a MALVERN-HPPS DLS (Malvern Instruments Ltd, Britain).

### Synthesis of the CNDs

The CNDs were prepared simply through one-pot hydrothermal treatment of *E. prolifera*. Dry smashed *E. prolifera* (2 g) were chopped very finely and dissolved in 120 mL of deionized water. The mixture was then transferred to a 200 mL Teflonlined autoclave and heated at 180 °C for 3, 6 or 10 h. The CNDs were collected by removing the suspended solids and large dots though centrifugation at 12000 rpm for 10 min. Different purification methods including filtration, centrifugation and ethanol sedimentation were tested to obtain high-quality CNDs. The purified solid-state CNDs were then dispersed in deionized water and then used for further characterization and applications.

### Cell imaging and ***in vitro*** cytotoxicity study for the as-prepared CNDs

The bioimaging test for the as-prepared CNDs was carried out using HeLa cells. The cells (10^6^ cells per sample) were plated onto 35 mm glass chamber slides, followed by the incubation in culture medium containing the CNDs at a concentration of 100 μg mL^−1^ for 24 h. All cells were washed with PBS buffer three times at room temperature to remove the free and physically absorbed CNDs. After that, the fluorescence images of the cells were taken using the LEICA TCS SP2 laser scanning confocal microscope (Leica Microsystems Heidelberg GmbH, Germany) with a 100 × oil immersion objective. The CNDs treated cells were excited by a 488 nm Ar laser^33^.

The cytotoxicity of the CNDs was evaluated using a standard MTT assay. The cells were treated with various concentrations of CNDs (20, 50, 100, 150, 200, 250, 300 μg mL^−1^, respectively) in fresh DMEM for 24 h. The cultured cells were added to DMEM containing MTT (10 mL, 5 mg mL^−1^) and further incubated at 5% CO_2_ and 37 ^◦^C for another 4 h. The MTT containing medium was then added with 100 μL DMSO to solubilize the formazan crystals precipitate. The cells incubated with PBS in the absence of CNDs were taken as the control group (viability defined as 100%), and six parallel samples incubated with CNDs were performed under the same conditions in each independent experiments. Finally, the absorption at 490 nm of each well was measured for calculation of the cell survival rates by an EL808 ultramicroplate reader (Bio-TEK Instrument, Inc., Winooski, VT, USA)^33^.

### Procedure for ion sensing and detection/removal of Fe^3+^ in seawater

All the metal ion stock solutions were prepared from their respective salts. Aqueous solutions of these metal ions were added into the CNDs suspension (20 μg mL^−1^) to reach all at the final concentration of 200 µM. Then the mixture was stirred and incubated for 10 min, respectively, followed by the fluorescence measurement. For Fe^3+^ sensing, similar steps were conducted. Typically, the fluorescence intensities of solution containing 100 μg mL^−1^ CNDs upon the addition of different concentrations of Fe^3+^ ranging from 0–1.7 mM were measured. The fluorescence spectra were recorded at an excitation wavelength of 370 nm accordingly^15^.

The tap sample was directly used after the acidified pretreatment by adding diluted hydrochloric acid. Fe^3+^ at different final concentrations (0, 2, 5, 10, 20, 50 μM) was spiked into the tap water containing 100 μg mL^−1^ CNDs, respectively. Then the PL intensity of the spiked sample was recorded accordingly. The decrease in PL intensity (ΔI = I_0_ − I_i_) was plotted against the spiked Fe^3+^ concentration to obtain the calibration curve. The concentration of Fe^3+^ in seawater water was calculated based on the absolute value of x-intercept from the calibration curve. The reproducibility of the analysis method was determined by measuring the relative standard deviations of replicate measurements (n = 3).

## Conclusions

Herein, *E. prolifera* was successfully converted to attractive fluorescent nanostructure CNDs by one-pot green hydrothermal process at 180 °C for 6 h followed by post-treatment of ethanol sedimentation. The QYs for the CNDs prepared before and after post-treatment reached 10.1% and 12.3%, respectively, which are higher than most of those obtained from other biomass as carbon sources for CNDs production. The as-prepared CNDs can be well-dispersed in aqueous medium with narrow size distribution and average size of 2.75 ± 0.12 nm as evidenced by TEM images. The as-prepared CNDs can be widely used in high-performance bioimaging, fiber staining as well as nanobioprobes for specific assay/removal of Fe^3+^. The CNDs was suggested to have great potential in further specific treatment of waste water containing excessive amounts of Fe^3+^. All in all, the ease synthesis and relatively high QYs of the as-obtained CNDs make this algal waste *E. prolifera* green and inexpensive benefit to the human being and nature. It also broadens the research in the synthesis of the CNDs and the application potentials in bio- and chemical-sensing. This work is of great importance to the improvement of the environmental issue and development of material science using nature waste materials.

## Electronic supplementary material

Supplementary Information
