# Supplementary material for: Enhanced-quantum yield sulfur/nitrogen co-doped fluorescent carbon nanodots produced from biomass Enteromorpha prolifera: synthesis, posttreatment, applications and mechanism study
Source: Sci Rep. 2017 Jul 3;7:4499. doi: 10.1038/s41598-017-04754-x (PMC5495774; doi:10.1038/s41598-017-04754-x)
Supplement: Supplementary file 1 — Supplementary Information [file 41598_2017_4754_MOESM1_ESM.doc]

Supporting Information for:

Enhanced-quantum yield sulfur/nitrogen co-doped fluorescent carbon nanodots produced from biomass *Enteromorpha prolifera*: synthesis, posttreatment, applications and mechanism study

YuanhongXu^a,*1^, Dan Li^b,1^, MengliLiu^a^, FushuangNiu^a^, Jingquan Liu^a,*^, Erkang Wang^b,*^

^a^Center for Micro/Nano Luminescent and Electrochemical Materials, College of Materials Science and Engineering; Institute for Graphene Applied Technology Innovation; Laboratory of Fiber Materials and Modern Textiles, the Growing Base for State Key Laboratory; Collaborative Innovation Center for Marine Biomass Fibers Materials and Textiles of Shandong Province, Qingdao University, Qingdao 266071, China

^b^State Key Laboratory of Electroanalytical Chemistry, Changchun Institute of Applied Chemistry, Chinese Academy of Sciences, Changchun, Jilin 130022, China

E-mail: yhxu@qdu.edu.cn (Y. Xu); jliu@qdu.edu.cn (J. Liu); ekwang@ciac.ac.cn (E. Wang)

^1^ YuanhongXu and Dan Li contributed equally to this work.


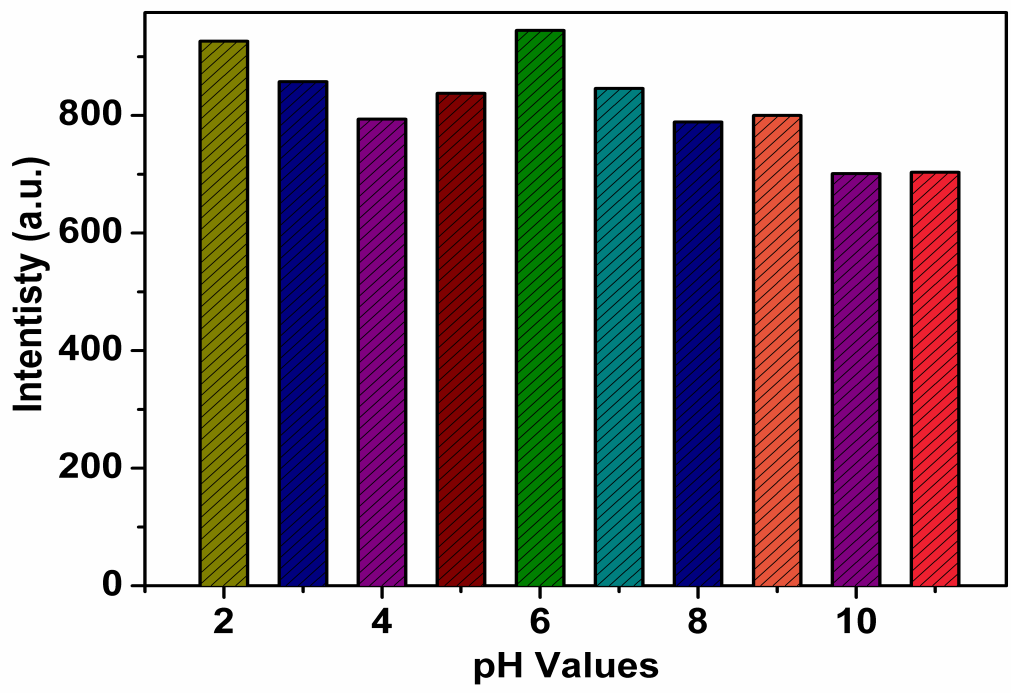


**Figure S1.** The effect of pH on the fluorescence intensity of the CNDs.


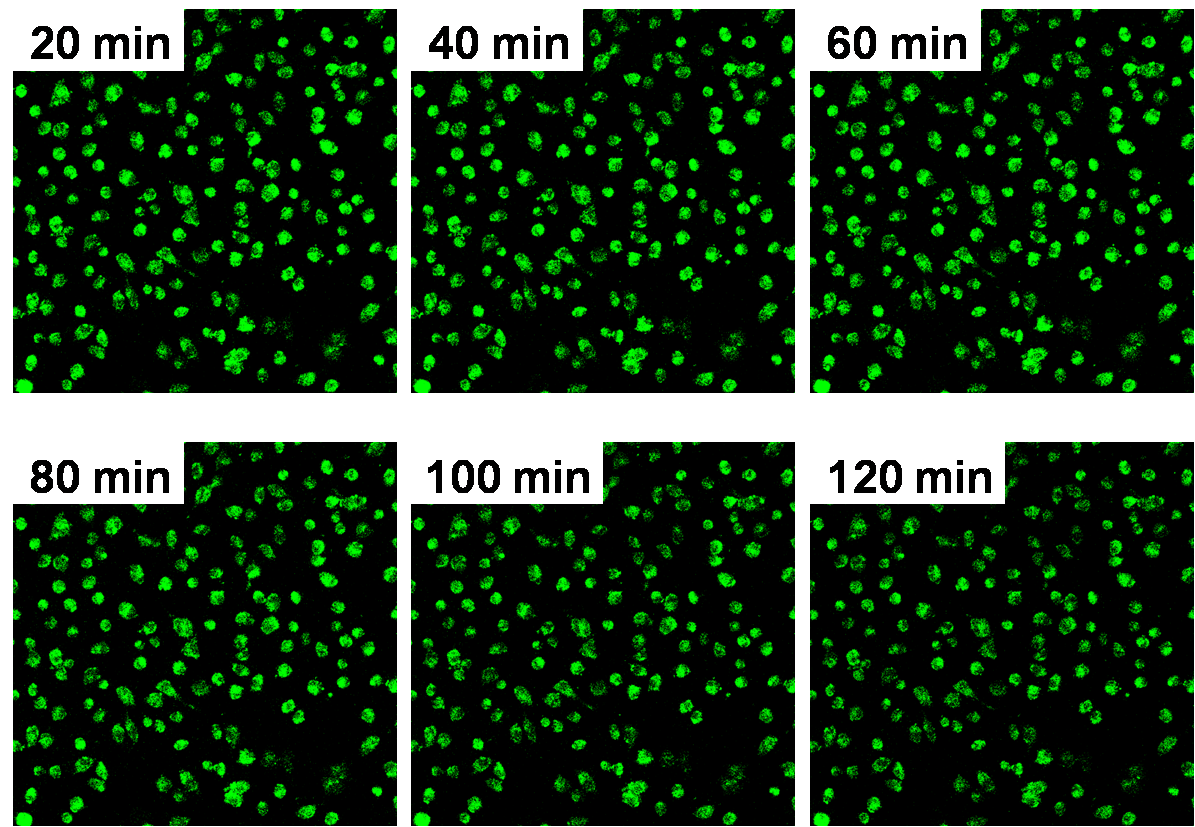


**Figure S2.** LSCM images showing time-dependent fluorescence signals of HeLa cells treated by the as-prepared CNDs.


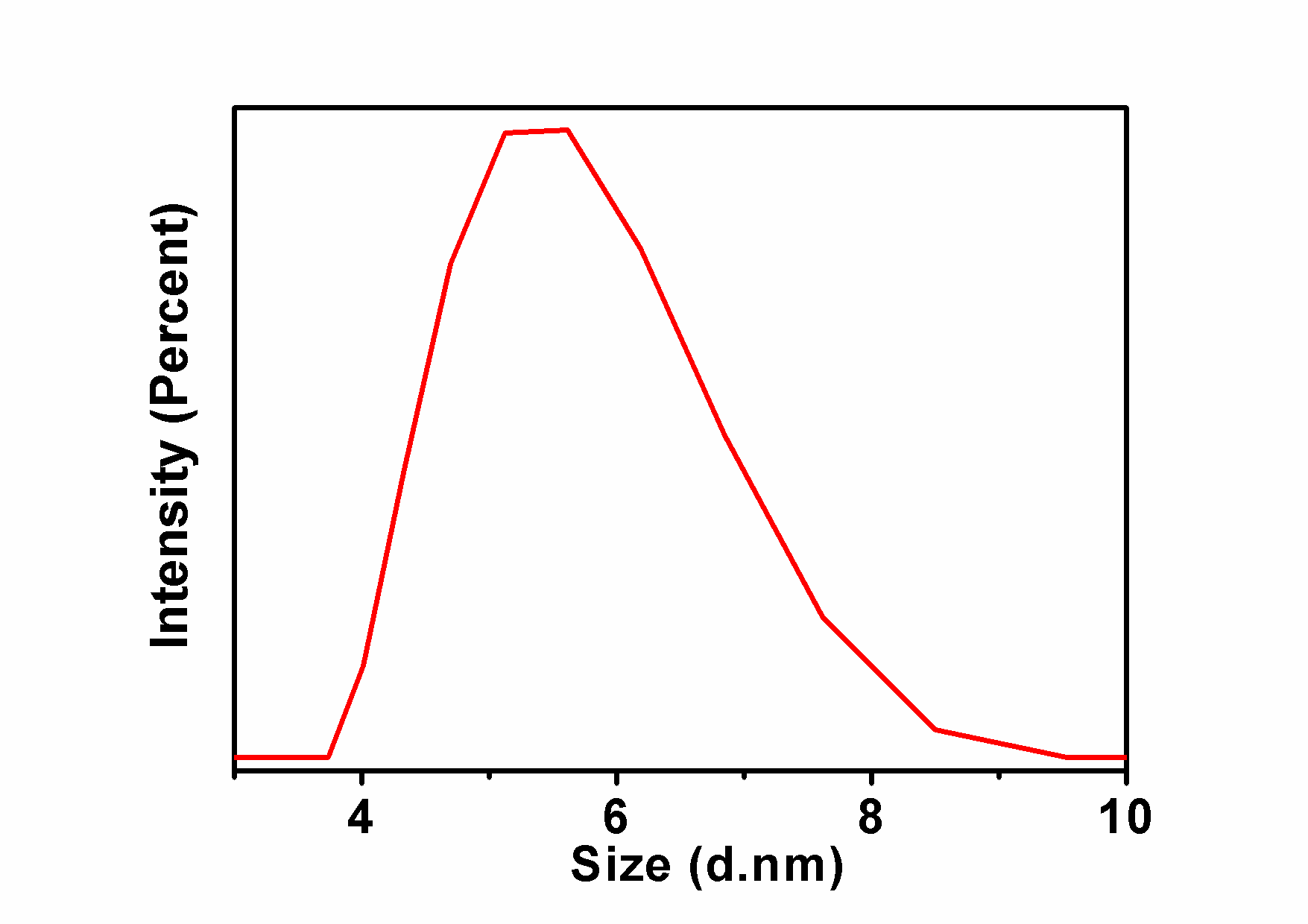


**Figure S3.** Size distribution of the as-prepared CNDs measured using dynamic light scattering (DLS).


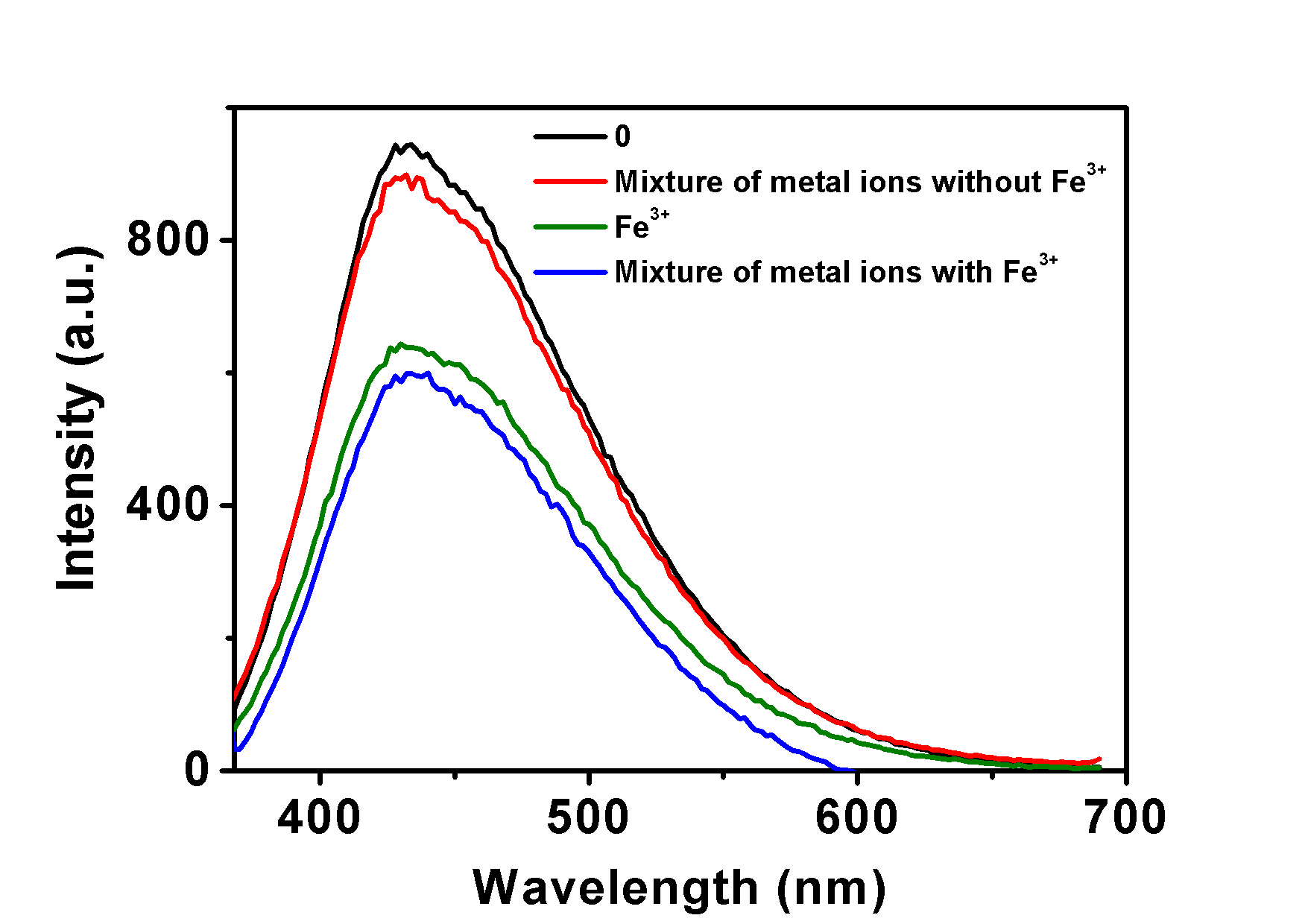


**Figure S4.** The fluorescence intensities of the as-prepared CNDs in the absence or presence of Fe^3+^ or mixture of different metal ions. All the tested metal ions were at 250 µM.


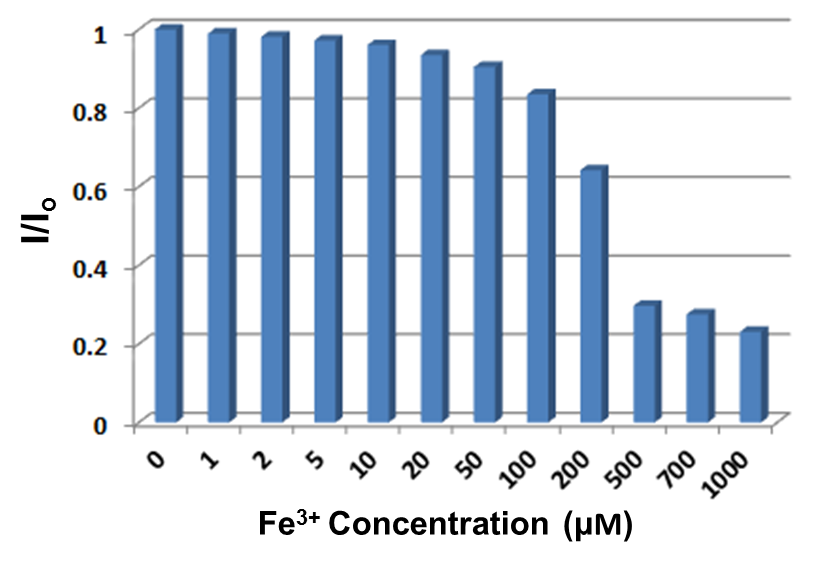


**Figure S5.** The change trend of the PL intensities of the CNDs suspension upon addition with different Fe^3+^ concentrations.

**Procedure of atomic absorption spectrophotometery (AAS) analysis**:

Atomic absorption spectrophotometer (AAS) equipped with hollow cathode lamp (HCL) was used for iron determination. The optimum conditions for AAS were applied as follow: wavelength 248.3 nm; HCL current 10 mA; acetylene flow rate 0.5 L/min; air flow rate 4.0 L/min; slit width 0.2 nm.

After the acid pretreatment by hydrochloric acid, iron (Fe^3+^) at different concentrations (0, 10, 20, 30 and 40 μM) was spiked into the tap water, respectively. The spiked water samples were determined by AAS method under the above analytical conditions. Concentrations of Fe^3+^ were calculated by the standard curve method [S1].

[S1] M. Yaman, G. Kaya, *Anal. Chim.Acta.***2005**, ***540***, 77-81.
